# The role of affective temperaments in bipolar disorder: The solid role of the cyclothymic, the contentious role of the hyperthymic, and the neglected role of the irritable temperaments

**DOI:** 10.1192/j.eurpsy.2023.16

**Published:** 2023-04-24

**Authors:** Elie G. Karam, Dahlia Saab, Salam Jabbour, Georges E. Karam, Elie Hantouche, Jules Angst

**Affiliations:** 1 Institute for Development, Research, Advocacy and Applied Care (IDRAAC), Beirut, Lebanon; 2 Department of Psychiatry and Clinical Psychology, St Georges University of Beirut, Beirut—Lebanon; 3 Department of Psychiatry and Clinical Psychology, St George Hospital University Medical Center, Beirut, Lebanon; 4 Anxiety & Mood Center, Paris, France; 5 University of Zurich, Zurich, Switzerland

**Keywords:** Affective temperament, bipolar disorder, cyclothymic temperament, hyperthymic temperament, irritable temperament

## Abstract

**Background:**

The aim of the present study is to evaluate the role of individual affective temperaments as clinical predictors of bipolarity in the clinical setting.

**Methods:**

The affective temperaments of 1723 consecutive adult outpatients presenting for various symptoms to a university-based mental health clinical setting were assessed. Patients were administered the Hypomania Checklist-32 and the Temperament Evaluation of Memphis, Pisa, Paris, and San Diego – Auto-questionnaire (TEMPS-A) and were diagnosed by psychiatrists according to the DSM-5 criteria. TEMPS-A scores were studied as both continuous and normalized categorical *z*-scores from a previously established nationwide study on the general population of Lebanon. Simple and multiple binary logistic regressions were done on patients who have any of the DSM-5 defined bipolar types, as a combined group or separately, versus patients without any bipolar diagnosis.

**Results:**

At the multivariable level and taking into account all temperaments, the irritable temperament is a consistent predictor of bipolar I and bipolar II disorders. Cyclothymic temperament also played a strong role in bipolarity but more decisively so in bipolar II and substance-induced bipolarity. The hyperthymic temperament had no role in bipolar I or bipolar II disorder.

## Background

Kraepelin posited that temperaments are “*rudiments of manic-depressive insanity*” or “fundamental states” that are “precursors which appear in early youth” and “continue to exist in the intervals between the attacks.” He identified four temperaments: the depressive, the manic, the irritable, and the cyclothymic. His observations, alongside reflections by major early German psychiatrists such as Schneider [1], greatly informed Akiskal’s work from the 1970s onwards, culminating in the construction of the TEMPS-A [2]. Akiskal and Mallya [3] initially identified four temperaments: the depressive (DT), cyclothymic (CT), irritable (IT), and hyperthymic (HT) temperaments and followed by the anxious temperament (AT) [4]. They were also conceptualized by Akiskal et al. to represent “attenuated phases of mood disorders” inseparably [5]. The notion that temperaments can be useful in predicting bipolar disorders sparked a plethora of research. Two main temperaments were targeted in bipolarity: the CT and the HT, and to a much lesser extent IT.

The CT, as measured by the TEMPS-A, like all temperaments is a lifelong trait characterized by frequent and rapid shifts between high and low moods and cognitive psychomotor perspectives, as well as instability in relationships. The relation of CT to bipolar disorders has been repeatedly demonstrated in several studies: patients with bipolar I and II had significantly higher scores on the TEMPS-A CT subscale compared to patients with major depressive disorder (MDD) [6–13] and crucially also to healthy controls [8, 14–18].

The HT was also actively researched among patients with bipolar disorder. The HT subscale is characterized, in the TEMPS-A, mostly through its positive characteristics such as cheerful mood, positive interpersonal relations, increased psychomotor activity, and cognitive capacities [19]. One early influential study proposed the HT as a diagnostic feature of bipolar II disorder [20], resulting in what we believe a subsequent overemphasis on the role of HT as a predictor of bipolarity [21]. However, a close inspection reveals that most studies found HT scores to be greater in patients with bipolarity when compared to those with MDD [22–27] but not to healthy controls [12, 14, 15, 17, 24–31].

The IT was delineated mostly through negative traits, as having a restless mood, feeling on edge, with angry outbursts and a tendency to ill-humored joking [21]. With the exception of one study [32], several publications found that patients with bipolarity have elevated IT scores compared to patients with depression and, also interestingly, to healthy controls [8, 9, 12–16, 26, 33, 34]. However, a common tacit assumption throughout the literature has been that CT and HT played the real and “logical” role rather than IT [6, 21, 35, 36].

There are several methodological issues in the studies of temperaments in bipolarity. First, many studies did not differentiate between bipolar I and bipolar II [32, 35, 37, 38]. The second issue is the lack of uniformity on how to quantify deviations of temperaments from the norm. Most studies used a wide variety of cut-offs [24, 39, 40] including a recurrent concept of “*prevalent*” or “*dominant*” temperaments, which were also variously defined and conceptualized [33, 41, 42]. Third and apart from one recent study [18], none had attempted to include all temperaments in a multivariable analysis to control for the well-established moderate to high correlations that are systematically found between temperaments [43–45]. Lastly, with the exception of one study [43], none of the studies relied on a solid such as a nationally representative reference of individual temperament scores; “normal scores” were based on nonrepresentative samples [45, 46].

As such, the aim of the present study is to address some of the limitations of the published literature in order to understand the importance of individual temperaments as clinical predictors of bipolar I and II disorders in a sample of outpatient participants.

## Methods

We conducted a cross-sectional study on a consecutive sample of 1,723 adult outpatients presenting between January 2014 and September 2019 for the first time for psychiatric consultation in the outpatient facilities of a university medical center (St Georges University Medical Center). Those with clear memory problems or illiteracy were excluded.

### Clinical diagnosis

The final clinical diagnosis was made through face-to-face interviews with all the patients and their accompanying relatives by two psychiatrists, based on a checklist of Diagnostic and Statistical Manual of Mental Disorders 5th edition (DSM-5) criteria [47]. All patients were also fully evaluated by highly experienced clinical assistants and any differences were resolved through active review of both individual and collateral reports. Furthermore, all patients with bipolarity were divided into the following DSM-5 subgroups: Bipolar I, Bipolar II, Other Specified Bipolar and Related Disorder, and Substance/Medication Induced Bipolar Disorder. Because the number of patients with medication/substance-induced mania (*n* = 7) and medication/substance-induced episodes characterized by mixed features (*n* = 2) were very small, these two categories were removed, restricting the Substance/Medication Induced Bipolar Disorder to those with medication/substance-induced hypomania (*n* = 39).

### Instruments

#### Temperament evaluation of Memphis, Pisa, Paris, and San Diego – Auto-questionnaire

The TEMPS-A is a 110-item well-established self-report measure developed to assess all the five temperaments DT, CT, IT, HT, and AT with good to excellent internal consistency [19]. The scale, used in this study, has been translated to several languages, including Lebanese-Arabic where it showed also good internal consistency on a nationally representative sample [43].

#### Hypomania checklist-32

The hypomania checklist-32 (HCL-32) is a widely used 32-item self-report measure developed to screen for bipolarity, based on the presence of manic symptoms throughout a person’s lifetime, using a Yes or No response format. It was designed to distinguish between participants who could be diagnosed with bipolar I or II disorder and those with MDD. The scale has an overall Cronbach alpha of 0.82 [48] and has been translated into several languages [49, 50]. A cut-off of 14 has been generally accepted as a cut-off for bipolarity. In our current clinical sample, using a cut-off of 14, the scale had a sensitivity of 0.81 and a specificity of 0.87 with bipolar I disorder and with bipolar II disorder, sensitivity and specificity were 0.82 and 0.87, respectively.

### Procedure

All adult participants who presented to the outpatient mental health facilities and completed the Lebanese Arabic TEMPS-A (for uniformity) (*N* = 1,723). Those who filled the English or the French TEMPS-A were excluded from the analysis (*N* = 1,652). This study was approved by the Institutional Review Board (IRB) committee of the SGHUMC Faculty of Medicine, University of Balamand, Lebanon (registered with the U.S. Office of Human Research Protections (OHRP) in the Department of Health and Human Services).

### Statistical analysis

Descriptive analyses using numbers and percentages for categorical variables and means with standard deviations (SD) for continuous variables were conducted. A distribution of temperament categorized *z*-scores by bipolar type was generated. Pearson correlation was used to test the correlation between the five temperaments. A logistic regression was conducted to investigate whether or not the TEMPS-A predicts Bipolarity, which is a dichotomous dependent variable. For significant predictors, an Odds Ratio greater than one indicates that the temperament is a risk factor and an Odds Ratio less than one indicates that the temperament is a protective factor. Simple and multiple binary logistic regressions were done on patients who have any of the bipolar types mentioned above, as a combined group, versus patients without any bipolar diagnosis. The same was done for those with Bipolar II disorder, Other Specified Bipolar and Related Disorder, and Substance/Medication Induced Bipolar Disorder, separately. In addition, simple and multiple binary logistic regressions were also conducted to compare a diagnosis of bipolar I disorder with a diagnosis of bipolar II disorder, across temperaments. In the Simple binary logistic regression, the five temperaments (DT, CT, HT, IT, and AT) were first tested separately with the dependent variable. Then in the multiple binary logistic regression, a model that contained the other temperaments with age and gender was performed. Temperaments were first taken as continuous scores, then were studied as *categorical z*-scores normalized using the mean and SD from the *general Lebanese population* [43]. The three categories of the various temperaments were: mean ± 1SD, >1SD to ≤2SD, and >2SD. The mean ± 1SD was set as the reference category. Crude and adjusted Odds Ratios (OR) with their 95% Confidence Intervals (CI) were generated. Analyses were conducted on the Statistical Package for the Social Sciences version 23.0 (SPSS).

## Results

### Description of the study population

The final sample consisted of a total of 1,723 patients, 369 of them with a confirmed DSM-5 bipolar diagnosis: Bipolar I (*n* = 52), Bipolar II (*n* = 176), Other Specified Bipolar and Related Disorder (*n* = 102), and Substance/Medication Induced Bipolar Disorder (*N* = 39) all analyzed separately. Total sample of 53.74% of 1,723 patients were females. In the total group of patients with any bipolar diagnosis, 53.93% were females; 47.09 in bipolar I and 56.82 in Bipolar II. The mean age of the total sample was 38.06 years (±14.85) and patients with a bipolar diagnosis were younger (see Supplementary Table S1).

### Correlation of temperaments

The correlations among temperaments were analyzed separately for all those with a bipolar I, bipolar II, any bipolar diagnosis, and those with a nonbipolar diagnosis (Supplementary Tables S2–S5, respectively).

### Predictors of all bipolar types

#### Temperaments as continuous scores

At the bivariate level, all continuous scores of temperaments, were significant predictors of patients with bipolarity (*n* = 369). At the multivariable level, all temperaments, except for AT, remained significantly associated with bipolarity. While increasing scores of IT, CT, and HT were associated with bipolarity, increasing scores of DT were reflective of lower chances of bipolarity (OR [95% CI]: 0.94[0.90–0.99]) (see Supplementary Table S6).

#### Temperaments as categorical normalized z-scores

At the bivariate level, when compared to temperament values which belonged to the category of mean ± 1SD, IT, CT, and AT were significant predictors of bipolarity. In the multivariable model, IT and CT increased the odds of bipolarity. At their highest (>2SD), CT was a stronger predictor than IT (OR [95% CI]: 3.84[2.52–5.87] vs. 2.55[1.72–3.79]) for CT and IT, respectively. In contrast, having a high score of DT (>2SD) decreased the odds of bipolarity (OR [95% CI]: 0.50[0.32–0.78]). HT and AT were not significant (see Table 1).

**Table 1.** All bipolars (*N* = 369) versus all nonbipolars (*N* = 1354): bivariate and multivariable regression analyses of affective temperaments as categorical normalized *z*-scores.

	Bivariate analysis	Multivariable analysis
Factor	Crude OR [95% CI]	Adjusted OR [95% CI]
Age	0.97 [0.96–0.98]*	0.98 [0.96–0.99]*
Gender (F)	1.01 [0.80–1.27]	1.32 [0.99–1.76]
IT (>1 to ≤2 SD)	2.16 [1.55–3.00]*	1.62 [1.08–2.43]
IT (>2SD)	4.51 [3.41–5.97]*	2.55 [1.72–3.79]*

CT (>1 to ≤2 SD)	2.75 [2.03–3.73]*	2.00 [1.35–2.96]*
CT (>2SD)	5.45 [4.04–7.36]*	3.84 [2.52–5.87]*

HT (>1 to ≤2 SD)	0.96 [0.62–1.49]	0.77 [0.47–1.27]
HT (>2SD)	0.37 [0.05–2.93]	0.24 [0.03–1.99]

AT (>1 to ≤2 SD)	1.96 [1.48–2.61]*	1.15 [0.79–1.67]
AT (>2SD)	2.48 [1.85–3.32]*	1.26 [0.83–1.93]

DT (>1 to ≤2 SD)	1.34 [1.03–1.75]*	0.78 [0.55–1.09]
DT (>2SD)	1.27 [0.94–1.73]	0.50 [0.32–0.78]*

*Note.* The temperaments reference normalized category is mean ± 1 SD.

* Significant at 0.05 level.

### Predictors of bipolar I

#### Temperaments as continuous scores

At the multivariable level, after adjusting for the presence of all temperaments as well as age and gender, only IT remained a significant predictor of patients with bipolar I disorder with adjusted OR of 1.19[1.09–1.29] (see Supplementary Table S7).

#### Temperaments as categorical normalized z-scores

In the multivariable model and compared to the national mean, the sole predictor of bipolar I was IT in its highest category (>2SD), OR: 4.13[1.72–9.96] (see Table 2).

**Table 2.** Bipolar I (*N* = 52) versus all nonbipolars (*N* = 1354): bivariate and multivariable regression analyses of affective temperaments as categorical normalized *z*-scores.

	Bivariate analysis	Multivariable analysis
Factor	Crude OR [95% CI]	Adjusted OR [95% CI]
Age	0.97 [0.95–0.99]*	0.98 [0.96–1.00]
Gender (F)	0.80 [0.46–1.39]	0.82 [0.43–1.54]

IT (>1 to ≤2 SD)	1.11 [0.45–2.73]	1.21 [0.44–3.32]
IT (>2SD)	3.92 [2.10–7.32]*	4.13 [1.72–9.96]*

CT (>1 to ≤2 SD)	2.40 [1.27–4.53]*	1.48 [0.66–3.33]
CT (>2SD)	1.90 [0.90–3.99]	0.82 [0.30–2.24]

HT (>1 to ≤2 SD)	0.71 [0.22–2.35]	0.62 [0.18–2.12]
HT (>2SD)	0	0

AT (>1 to ≤2 SD)	1.89 [0.96–3.72]	0.90 [0.39–2.08]
AT (>2SD)	2.20 [1.10–4.42]	1.03 [0.40–2.61]

DT (>1 to ≤2 SD)	1.59 [0.86–2.96]	1.34 [0.65–2.78]
DT (>2SD)	1.25 [0.59–2.66]	0.57 [0.20–1.64]

*Note.* The temperaments reference normalized category is mean ± 1 SD.

* Significant at 0.05 level.

### Predictors of bipolar II

#### Temperaments as continuous scores

All temperaments, with the exception of AT, remained significant predictors of bipolar II at the multivariable level: while higher scores of IT, CT, and HT increased the odds of bipolarity, higher scores of DT lowered the odds of bipolar II (OR [95% CI]: 0.93[0.87–0.99]) (see Supplementary Table S8).

#### Temperaments as categorical normalized z-scores

Table 3 summarizes the results of the crude and adjusted ORs of temperaments in predicting bipolar II as compared to the reference category of the *national* mean ± 1SD. At the multivariable level, clearly both IT and CT had an important role in predicting bipolar II (at levels >1SD to ≤2SD and at >2SD). At its highest (>2SD), CT was a stronger predictor of bipolar II than IT (OR [95% CI]: 4.38 [2.44–7.86] vs. 3.37[1.88–6.05]. DT (at >2SD) looks to have a protective role for bipolar II compared to patients without bipolarity (OR [95% CI]: 0.41[0.22–0.74]).

**Table 3.** Bipolar II (*N* = 176) versus all nonbipolars (*N* = 1354): bivariate and multivariable regression analyses of affective temperaments as categorical normalized *z*-scores.

	Bivariate analysis	Multivariable analysis
Factor	Crude OR [95% CI]	Adjusted OR [95% CI]
Age	0.96 [0.94–0.97]*	0.96 [0.95–0.98]*
Gender (F)	1.14 [0.83–1.56]	1.79 [1.20–2.67]*

IT (>1 to ≤2 SD)	3.16 [1.96–5.10]*	2.53 [1.41–4.56]*
IT (>2SD)	6.69 [4.43–10.11]*	3.37 [1.88–6.05]*

CT (>1 to ≤2 SD)	3.55 [2.25–5.60]*	1.95 [1.11–3.45]*
CT (>2SD)	8.07 [5.23–12.46]*	4.38 [2.44–7.86]*

HT (>1 to ≤2 SD)	1.42 [0.84–2.41]	1.10 [0.61–2.00]
HT (>2SD)	0.81 [0.10–6.43]	0.50 [0.06–4.22]

AT (>1 to ≤2 SD)	2.26 [1.52–3.38]*	1.06 [0.63–1.79]
AT (>2SD)	3.04 [2.03–4.55]*	1.52 [0.86–2.69]

DT (>1 to ≤2 SD)	1.25 [0.87–1.79]	0.67 [0.43–1.06]
DT (>2SD)	1.18 [0.78–1.80]	0.41 [0.22–0.74]*

*Note.* The temperaments reference normalized category is mean ± 1 SD.

* Significant at 0.05 level.

### Predictors of patients diagnosed with other specified bipolar and related disorder

#### Temperaments as continuous scores

In the multivariable analysis, only IT (OR [95% CI]: 1.07[1.01–1.14]), CT (OR [95% CI]: 1.15[1.08–1.22]), and HT(OR [95% CI]: 1.07 [1.02–1.13]) remained predictors of the diagnosis of Other Specified Bipolar and Related Disorder (*n* = 102) (see Supplementary Table S9).

#### Temperaments as categorical normalized z-scores

At the multivariable level, only CT (at >1SD to ≤2SD and at >2SD) was a predictor (OR [95% CI]: 2.14[1.06–4.30] and OR [95%CI]: 5.17[2.51–10.64], respectively) of Other Bipolar Disorder and Related Disorder (see Supplementary Table S10).

### Predictors of substance/medication-induced bipolar disorder-hypomanic episodes

#### Temperaments as continuous scores

In the multivariable analysis, CT remained the only predictor (OR [95% CI]: 1.15[1.04–1.27]) of Substance/Medication-Induced Bipolar Disorder, all of the included had hypomanic episodes (*n* = 39) (see Supplementary Table S11).

#### Temperaments as categorical normalized z-scores

At the multivariate level, as compared to the reference category of the *normal* mean ± 1SD, *r*, only CT (>2SD) was a predictor of Substance/Medication-Induced Bipolar Disorder (OR [95% CI]: 6.46[2.04–20.49]) (see Table 4).

**Table 4.** Medication/substance-induced bipolar disorder – hypomanic episodes (*N*-39) versus all nonbipolars (*N* = 1354): bivariate and multivariable regression analyses of affective temperaments as categorical normalized *z*-scores.

	Bivariate analysis	Multivariable analysis
Factor	Crude OR [95% CI]	Adjusted OR [95% CI]
Age	1.00 [0.98–1.02]	1.00 [0.98–1.03]
Gender (F)	1.38 [0.72–2.66]	1.43 [0.70–2.94]

IT (>1 to ≤2 SD)	1.17 [0.44–3.12]	0.77 [0.24–2.47]
IT (>2SD)	3.33 [1.64–6.77]*	1.85 [0.68–5.01]

CT (>1 to ≤2 SD)	2.62 [1.09–6.27]*	2.69 [0.88–8.23]
CT (>2SD)	5.69 [2.52–12.83]*	6.46 [2.04–20.49]*

HT (>1 to ≤2 SD)	0.59 [0.14–2.50]	0.54 [0.12–2.40]
HT (>2SD)	0	0

AT (>1 to ≤2 SD)	1.35 [0.60–3.04]	0.84 [0.31–2.28]
AT (>2SD)	2.39 [1.12–5.09]*	1.13 [0.39–3.29]

DT (>1 to ≤2 SD)	1.67 [0.79–3.55]	0.79 [0.32–1.94]
DT (>2SD)	2.10 [0.95–4.64]	0.80 [0.29–2.22]

*Note.* The temperaments reference normalized category is mean ± 1 SD.

* Significant at 0.05 level.

### Predictors of bipolar I versus bipolar II disorder

#### Temperaments as continuous scores

At both the bivariate and multivariate levels, when taken as continuous scores, only CT was able to differentiate patients with bipolar II from those with bipolar I disorder (OR [95% CI]: 1.16[1.07–1.25] and OR [95%CI]: 1.21[1.08–1.35] for the bivariate and multivariate analysis, respectively) (see Supplementary Table S12).

#### Temperaments as categorical normalized z-scores

At the bivariate level, as compared to the *reference* category of mean ± 1SD, both IT (>1 to ≤2SD) and CT (>2SD) significantly predicted patients with bipolar II disorder over those with bipolar I disorder. At the multivariate level, only CT (>2SD) significantly differentiated those with a bipolar II diagnosis from Bipolar I (OR [95%CI]: 4.59[1.43–14.76]) (see Table 5).

**Table 5.** Bipolar II (*N* = 176) versus bipolar I (*N* = 52): bivariate and multivariable regression analyses of affective temperaments as categorical normalized *z*-scores.

	Bivariate analysis	Multivariable analysis
Factor	Crude OR [95% CI]	Adjusted OR [95% CI]
Age	0.99 [0.96–1.01]	0.98 [0.95–1.01]
Gender (F)	1.42 [0.76–2.64]	1.68 [0.81–3.51]

IT (>1 to ≤2 SD)	2.84 [1.05–7.72]*	2.19 [0.69–6.92]
IT (>2SD)	1.71 [0.83–3.52]	1.13 [0.40–3.19]

CT (>1 to ≤2 SD)	1.48 [0.69–3.17]	1.34 [0.49–3.68]
CT (>2SD)	4.26 [1.84–9.84]*	4.59 [1.43–14.76]*

HT (>1 to ≤2 SD)	2.00 [0.56–7.09]	1.57 [0.40–6.12]
HT (>2SD)	∞ [0]	∞ [0]

AT (>1 to ≤2 SD)	1.20 [0.56–2.58]	0.88 [0.34–2.32]
AT (>2SD)	1.38 [0.63–3.01]	1.17 [0.39–3.47]

DT (>1 to ≤2 SD)	0.78 [0.39–1.57]	0.44 [0.18–1.06]
DT (>2SD)	0.95 [0.41–2.19]	0.60 [0.18–1.98]

*Note.* The temperaments reference normalized category is mean ± 1 SD.

* Significant at 0.05 level.

### The case of the hyperthymic temperament

The hyperthymic temperament (HT) was not associated with a diagnosis of bipolarity, neither when patients with bipolarity were grouped together into one category nor when patients with bipolar I and II disorders were considered separately when using normalized categorical *z*-scores (>1SD to ≤2SD and > 2SD) of temperaments. This contrasts with findings when using continuous temperament scores. However, and quite importantly, the increased odds of HT in the continuous scores’ calculations were in fact restricted only to the bracket of mean ± 1SD compared to the mean. Again, this was true whether we looked at all patients with bipolarity as a group or when bipolar I and II disorders were considered separately. It is important to note however that by definition, the range ± 1SD is the “normal” range and not a truly elevated value (which should start at least at above 1SD), indicating that this HT finding on continuous score does not have any real significance.

## Discussion

Since Kraepelin’s early formulation and Akiskal’s revival and elaboration on the role of affective temperaments as fundamental states or *formes frustres* of bipolar disorders [11, 51, 52], we have come to understand and explore affective temperaments not only as part of the normal variations of human emotions and behaviors, but also as possible attenuated forms of bipolarity. Our present study addresses the previously published research on the role of temperaments, and more specifically the cyclothymic (CT), hyperthymic (HT), and irritable temperaments (IT), as clinical predictors of bipolar disorders in outpatients.

There are two major reasons for inconsistencies in the literature regarding the role of temperaments in bipolarity. First, and across cultures, temperaments were universally correlated with each other, in both clinical and nonclinical populations [17, 43–45]. The same was true in our present study: correlations among temperaments were solid whether looking at patients with bipolarity or not, further emphasizing the necessity of controlling for them. The second reason for the inconsistencies in the published literature lies in the fact that measurements of temperaments did not use *normalized* temperament scores. Therefore, to better understand the role of specific temperaments, we used normative data from our national study [43] in addition to multivariable regression analyses which controlled for intertemperamental correlations.

The irritable and cyclothymic temperaments played important roles among patients diagnosed with any bipolar diagnosis. At the multivariable level, and adjusting for all temperaments, IT was a significant predictor of a bipolar I diagnosis. In bipolar II both IT and CT were predictors, with CT being the stronger predictor of the two. CT was the only significant predictor of a diagnosis of Other Specified and Related Disorder and those who developed hypomanic episodes induced by substances/medications. These results, quite importantly, underlie the very important but neglected role of IT in bipolar I. High DT was protective against a diagnosis of bipolar II disorder and AT did not play a role in either subtype.

Finally, we could not demonstrate any role for HT in predicting any bipolar diagnosis, at the multivariate level. When HT scores were considered as continuous scores, we initially found HT to be a predictor of bipolarity when all bipolarity patients were grouped together and also in different bipolar subtypes, a finding similar to some studies [8, 16]. However, as we highlighted above in the section “Results,” when we looked closer at this, we found that the predictive role of HT was limited only to the normal ranges of 0–1 SD above the mean. Thus, HT cannot be considered statistically a predictor since, by definition, the range of ±1 SD refers to the normal levels. In addition, when looking closer at this issue from a different angle, we checked again the numbers from our national study on the general population from which the normalized scores were constructed [43]: we found that 19.60% of the general population had HT scores above one standard deviation whereas only 7.90% of all patients with bipolarity in our present clinical population had HT scores above 1SD. In addition, and in contrast, 74 and 76.4% of patients with any bipolar diagnosis in this study had scores of IT and CT, respectively, above 1SD, in comparison to around 15% in the general population (see Table 6). In other words, the findings from our national study mirror the findings from our present study of the outpatient clinical population.

**Table 6.** Affective temperaments: comparison of national sample to outpatient clinical sample.

	Hyperthymic temperament					
	*N*	<−2SD (%)	≥−2SD, <−1SD (%)	Mean ± 1 SD (%)	>1SD; ≤2SD (%)	>2SD (%)
Total Lebanese population: national study	1279	5.00	11	65.10	19.60	0.00
Clinical population: All bipolars	369	1.10	14.1	77.0	7.60	0.30
Irritable temperament						
Total Lebanese population: National study	1279	0.00	0.00	86.10	7.70	6.20
Clinical population: All bipolars	369	0.50	0.30	25.20	21.4	52.6
Cyclothymic temperament						
Total Lebanese population: National study	1279	0.00	14	70.30	11.20	4.40
Clinical population: All bipolars	369	0.30	0.30	23	32.2	44.2

The strengths of our study lie in addressing many of the limitations from previous studies. One important strength is that we carried multivariable regression analyses to control for the effect of other temperaments when zooming on the effect of each temperament and taking into consideration the known intercorrelations across all the five temperaments with gender and age entered as covariates. A second equally important strength is the use of normative temperament scores as a reference based on a nationally representative sample of the general Lebanese population [43]. This to our knowledge is the only study to have relied on nationally representative *z*-scores. By comparing patients’ scores to normative scores, temperaments may be examined through a lens that situates participants within the total population and not simply with other highly selected groups. Another strength is that we analyzed bipolar I and II disorders separately since they have been shown to have distinct clinical presentations [53, 54].

Nevertheless, the study carries some limitations. Formal structured interviews were not used; yet, the diagnoses were established by highly experienced clinicians and experienced physician assistants, who strictly followed the DSM-5 criteria. Whenever any uncertainties arose, the differences in each case were resolved through discussion and review of evidence from patients and accompanying relatives alike. Furthermore, all patients completed the HCL-32, the sensitivity and specificity scores of which compare very well to other published studies [49, 50]. However, we do recognize that structured interviews are helpful in establishing benchmarks and comparability across studies, despite their inherent limitations in underdiagnosing bipolar II disorder [55]. Since our recruitment method relied only on an outpatient sample, another potential limitation is that inpatients with bipolarity might have different profiles and that our population is not representative of all of patients with bipolarity. Additionally, while state effects on the TEMPS-A self-rating might be present [45, 56], our study’s clinical implications apply only to outpatients coming for treatment rather than euthymic patients: this mirrors clinical reality, since patients who present to the clinic are, rarely, if ever, euthymic but are, due to their presence in the clinic, quite likely to be experiencing symptoms. Furthermore, an important limitation specific to this study is the relatively smaller number of patients with bipolar I disorder (*N* = 52), who typically come to the ER and are admitted while the number of bipolar II is much larger (*N* = 176). One could also argue also that some of the patients with bipolar II disorder in our sample might convert to bipolar I disorder, and thus affect the predictive value of our results. This seems unlikely since only a small proportion (5%) of those with bipolar II disorder have been reported to convert to bipolar I disorder [57]. Finally, our findings might differ between countries as normative scores of temperaments might differ across cultures.

In conclusion, our study showed that IT was a consistent predictor of both bipolar I and II, playing a more prominent role in bipolar I disorder. CT also played quite a strong role but more decisively in bipolar II disorder and medication/substance-induced bipolar disorder. It is important to note that our results do not negate the probable role of CT also in bipolar I disorder, as we had found CT to be a robust predictor in our bivariate analyses, CT’s role may have been more pronounced had we had a much larger sample of patients diagnosed with bipolar I. Thus, this finding needs to be replicated. With the established underdiagnoses of bipolarity (especially in bipolar II disorder) in most epidemiological studies [55], the incorporation of temperaments into the assessment of patients and research participants alike is likely to help us detect the presence of bipolarity more readily and quite importantly help us in our quest to understand their genesis. Finally, ideally only prospective studies, evaluating temperaments before the onset of any mental disorder, would offer the conclusive answers to these issues.

## Supplementary material

For supplementary material accompanying this paper visit http://doi.org/10.1192/j.eurpsy.2023.16. click here to view supplementary material
