# Supplementary material for: The role of affective temperaments in bipolar disorder: The solid role of the cyclothymic, the contentious role of the hyperthymic, and the neglected role of the irritable temperaments
Source: Eur Psychiatry. 2023 Apr 24;66(1):e37. doi: 10.1192/j.eurpsy.2023.16 (PMC10228355; doi:10.1192/j.eurpsy.2023.16)
Supplement: Supplementary file 1 [file S0924933823000160sup001.docx]

**The Role of Affective Temperaments in Bipolar Disorder: The Solid Role of the Cyclothymic, the Contentious Role of the Hyperthymic, and the Neglected Role of the Irritable Temperaments (Supplementary Tables 1 to 12)**

**Table S1: Diagnoses of the outpatient population**

|  | Total | Male | Female | Age (years) |
| --- | --- | --- | --- | --- |
|  | N | N (%) | N (%) | Mean (SD) |
| Bipolar I | 52 | 27 (51.92) | 25 (48.08) | 33.25 (13.86) |
| Bipolar II | 176 | 76 (43.18) | 100 (56.82) | 31.22 (10.42) |
| Other Specified Bipolar and Related Disorder | 102 | 52 (51.00) | 50 (49.00) | 33.06 (12.22) |
| Medication Induced Hypomania | 39 | 15 (38.46) | 24 (61.54) | 38.90 (15.39) |
| All Bipolar | 369 | 170 (46.07) | 199 (53.93) | 32.83 (12.20) |
| Non-Bipolar | 1354 | 627 (46.34) | 726 (53.66) | 39.48 (15.19) |
| Total Patient sample | 1723 | 797 (46.26) | 925 (53.74) | 38.06 (14.85) |

**Table S2: Correlation matrix of temperaments in patients with bipolar I (N=52)**

|  | | IT score | AT score | HT score | DT score | CT score |
| --- | --- | --- | --- | --- | --- | --- |
| IT score | Pearson Correlation |  | **.49^**^** | **.05** | **.0.39^**^** | **.62^**^** |
|  | Sig. (2-tailed) |  | <.001 | .718 | .004 | <.001 |
| AT score | Pearson Correlation |  |  | **-.16** | **.58^*^**^*^ | **.45^**^** |
|  | Sig. (2-tailed) |  |  | .245 | <.001 | .001 |
| HT score | Pearson Correlation |  |  |  | **-.40^**^** | **-.04** |
|  | Sig. (2-tailed) |  |  |  | .004 | .793 |
| DT score | Pearson Correlation |  |  |  |  | **.38^**^** |
|  | Sig. (2-tailed) |  |  |  |  | .005 |

*: correlation is significant at 0.05 level (2-tailed)

**: correlation is significant at 0.01 level (2-tailed)

**Table S3: Correlation matrix of temperaments in patients with bipolar II (N=176)**

|  | | IT score | AT score | HT score | DT score | CT score |
| --- | --- | --- | --- | --- | --- | --- |
| IT score | Pearson Correlation |  | **.48^**^** | **.08** | **.30^**^** | **.47^**^** |
|  | Sig. (2-tailed) |  | <.001 | .316 | <.001 | <.001 |
| AT score | Pearson Correlation |  |  | **-.05** | **.44^*^**^*^ | **.52^**^** |
|  | Sig. (2-tailed) |  |  | .491 | <.001 | <.001 |
| HT score | Pearson Correlation |  |  |  | **-.24^**^** | **-.04** |
|  | Sig. (2-tailed) |  |  |  | .001 | .623 |
| DT score | Pearson Correlation |  |  |  |  | **.40^**^** |
|  | Sig. (2-tailed) |  |  |  |  | <.001 |

*: correlation is significant at 0.05 level (2-tailed)

**: correlation is significant at 0.01 level (2-tailed)

**Table S4: Correlation matrix of temperaments in all patients with bipolar disorder (N=369)**

|  | | IT score | AT score | HT score | DT score | CT score |
| --- | --- | --- | --- | --- | --- | --- |
| IT score | Pearson Correlation |  | **.47^**^** | **.14*** | **.31^**^** | **.52^**^** |
|  | Sig. (2-tailed) |  | <.001 | .008 | <.001 | <.001 |
| AT score | Pearson Correlation |  |  | **-.02** | **.53^*^**^*^ | **.50^**^** |
|  | Sig. (2-tailed) |  |  | .778 | <.001 | <.001 |
| HT score | Pearson Correlation |  |  |  | **-.20^**^** | **.08** |
|  | Sig. (2-tailed) |  |  |  | <.001 | .123 |
| DT score | Pearson Correlation |  |  |  |  | **.45^**^** |
|  | Sig. (2-tailed) |  |  |  |  | <.001 |

*: correlation is significant at 0.05 level (2-tailed)

**: correlation is significant at 0.01 level (2-tailed)

**Table S5: Correlation matrix of temperaments in all patients without bipolarity (N=1354)**

|  | | IT score | AT score | HT score | DT score | CT score |
| --- | --- | --- | --- | --- | --- | --- |
| IT score | Pearson Correlation |  | **.56^**^** | **.05** | **.0.45^**^** | **.64^**^** |
|  | Sig. (2-tailed) |  | <.001 | .065 | <.001 | <.001 |
| AT score | Pearson Correlation |  |  | **-.14^**^** | **.60^*^**^*^ | **.60^**^** |
|  | Sig. (2-tailed) |  |  | <.001 | <.001 | <.001 |
| HT score | Pearson Correlation |  |  |  | **-.26^**^** | **-.02^*^** |
|  | Sig. (2-tailed) |  |  |  | <.001 | .530 |
| DT score | Pearson Correlation |  |  |  |  | **.57^**^** |
|  | Sig. (2-tailed) |  |  |  |  | <.001 |

*: correlation is significant at 0.05 level (2-tailed)

**: correlation is significant at 0.01 level (2-tailed)

**Table S6: Bivariate and Multivariable Regression Analyses- ALL BP (bipolar=369 and non-bipolar N=1354; temps continuous scores)**

|  | Bivariate Analysis | | | Multivariable Analysis | | |
| --- | --- | --- | --- | --- | --- | --- |
| factor | crude OR | 95% CI lower | 95% CI upper | adjusted OR | 95% CI lower | 95% CI upper |
| Age | 0.97* | 0.96 | 0.97 | 0.98* | 0.97 | 0.99 |
| Gender (F) | 1.01 | 0.80 | 1.27 | 1.36* | 1.04 | 1.78 |
| IT | 1.20* | 1.16 | 1.23 | 1.10* | 1.06 | 1.14 |
| CT | 1.21* | 1.17 | 1.24 | 1.17* | 1.12 | 1.21 |
| HT | 1.09* | 1.06 | 1.10 | 1.07* | 1.04 | 1.11 |
| AT | 1.08* | 1.06 | 1.10 | 0.99 | 0.97 | 1.03 |
| DT | 1.07* | 1.03 | 1.10 | 0.94* | 0.90 | 0.99 |

*: significant at 0.05 level

**Table S7: Bivariate and Multivariable Regression Analyses- Bipolar I (N=52; temps continuous scores)**

|  | Bivariate Analysis | | | Multivariable Analysis | | |
| --- | --- | --- | --- | --- | --- | --- |
| factor | crude OR | 95% CI lower | 95% CI upper | adjusted OR | 95% CI lower | 95% CI upper |
| Age | 0.97* | 0.95 | 0.99 | 0.98 | 0.96 | 1.00 |
| Gender (F) | 0.80 | 0.46 | 1.39 | 1.32 | 0.72 | 2.40 |
| IT | 1.20* | 1.13 | 1.28 | 1.19* | 1.09 | 1.29 |
| CT | 1.21* | 1.06 | 1.19 | 1.04 | 0.96 | 1.13 |
| HT | 1.10* | 1.03 | 1.17 | 1.07 | 1.00 | 1.15 |
| AT | 1.05* | 1.01 | 1.10 | 1.00 | 0.94 | 1.07 |
| DT | 1.02 | 0.95 | 1.10 | 0.92 | 0.82 | 1.03 |

*: significant at 0.05 level

**Table S8: Bivariate and Multivariable Regression Analyses- Bipolar II (N=176; temps continuous scores)**

|  | Bivariate Analysis | | | Multivariable Analysis | | |
| --- | --- | --- | --- | --- | --- | --- |
| factor | crude OR | 95% CI lower | 95% CI upper | adjusted OR | 95% CI lower | 95% CI upper |
| Age | 0.96* | 0.94 | 0.97 | 0.97* | 0.96 | 0.98 |
| Gender (F) | 1.14 | 0.83 | 1.56 | 1.64* | 1.14 | 2.38 |
| IT | 1.22* | 1.18 | 1.27 | 1.10* | 1.04 | 1.16 |
| CT | 1.28* | 1.22 | 1.33 | 1.23* | 1.16 | 1.30 |
| HT | 1.10* | 1.06 | 1.14 | 1.08* | 1.04 | 1.13 |
| AT | 1.10* | 1.07 | 1.13 | 1.00 | 0.96 | 1.04 |
| DT | 1.08* | 1.03 | 1.12 | 0.93* | 0.87 | 0.99 |

*: significant at 0.05 level

**Table S9: Bivariate and Multivariable Regression Analyses- Other Specified Bipolar and Related Disorder (N=102; temps continuous scores)**

|  | Bivariate Analysis | | | Multivariable Analysis | | |
| --- | --- | --- | --- | --- | --- | --- |
| Factor | crude OR | 95% CI lower | 95% CI upper | adjusted OR | 95% CI lower | 95% CI upper |
| Age | 0.97* | 0.95 | 0.98 | 0.98* | 0.96 | 1.00 |
| Gender (F) | 0.83 | 0.56 | 1.24 | 1.23 | 0.79 | 1.91 |
| IT | 1.16* | 1.11 | 1.21 | 1.07* | 1.01 | 1.14 |
| CT | 1.17* | 1.12 | 1.23 | 1.15* | 1.08 | 1.22 |
| HT | 1.09* | 1.04 | 1.14 | 1.07* | 1.02 | 1.13 |
| AT | 1.05* | 1.02 | 1.09 | 0.99 | 0.95 | 1.04 |
| DT | 1.04 | 0.99 | 1.10 | 0.94 | 0.87 | 1.02 |

*: significant at 0.05 level

**Table S10: Bivariate and Multivariable Regression Analyses- Other Specified Bipolar and Related Disorder (N=102; temps as categorical normalized z-scores) ^+^**

|  | Bivariate Analysis | Multivariable Analysis |
| --- | --- | --- |
| Factor | crude OR [95% CI] | adjusted OR [95% CI] |
| Age | 0.97 [0.95-0.98]* | 0.98 [0.96-0.99]* |
| Gender (F) | 0.83 [0.56-1.24] | 1.24 [0.76-2.03] |
|  | | |
| IT (>1 to ≤ 2 SD) | 2.05 [1.19-3.54]* | 1.22 [0.61-2.41] |
| IT (>2SD) | 3.00 [1.86-4.85]* | 1.48 [0.76-2.90] |
|  | | |
| CT (>1 to ≤ 2 SD) | 2.11 [1.23-3.62]* | 2.14 [1.06-4.30]* |
| CT (>2SD) | 4.74 [2.88-7.80]* | 5.17 [2.51-10.64]* |
|  | | |
| HT (>1 to ≤ 2 SD) | 0.49 [0.18-1.36] | 0.32 [0.10-1.07] |
| HT (>2SD) | 0 | 0 |
|  | | |
| AT (>1 to ≤ 2 SD) | 1.83 [1.13-2.97]* | 1.36 [0.73-2.53] |
| AT (>2SD) | 1.88 [1.13-3.15]* | 1.07 [0.51-2.26] |
|  | | |
| DT (>1 to ≤ 2 SD) | 1.29 [0.81-2.05] | 0.77 [0.43-1.38] |
| DT (>2SD) | 1.19 [0.70-2.05] | 0.56 [0.26-1.19] |

^+^: the temperaments reference normalized category is mean ± 1 SD

*: significant at 0.05 level

**Table S11: Bivariate and Multivariable Regression Analyses- Medication/substance induced hypomania (N=39; temps continuous scores)**

|  | Bivariate Analysis | | | Multivariable Analysis | | |
| --- | --- | --- | --- | --- | --- | --- |
| factor | crude OR | 95% CI lower | 95% CI upper | adjusted OR | 95% CI lower | 95% CI upper |
| Age | 1.00 | 0.98 | 1.02 | 1.01 | 0.98 | 1.03 |
| Gender (F) | 1.38 | 0.72 | 2.66 | 1.74 | 0.85 | 3.52 |
| IT | 1.17* | 1.09 | 1.25 | 1.08 | 0.98 | 1.20 |
| CT | 1.20* | 1.11 | 1.30 | 1.15* | 1.04 | 1.27 |
| HT | 1.04 | 0.96 | 1.11 | 1.05 | 0.97 | 1.14 |
| AT | 1.10* | 1.04 | 1.17 | 1.00 | 0.93 | 1.08 |
| DT | 1.15* | 1.05 | 1.26 | 1.03 | 0.91 | 1.16 |

*: significant at 0.05 level

**Table S12: Bivariate and Multivariable Regression Analyses- Comparing bipolar II to bipolar I (reference) bipolar I N=52 and bipolar II N=176 (temps continuous scores)**

|  | Bivariate Analysis | | | Multivariable Analysis | | |
| --- | --- | --- | --- | --- | --- | --- |
| Factor | crude OR | 95% CI lower | 95% CI upper | adjusted OR | 95% CI lower | 95% CI upper |
| Age | 0.99 | 0.96 | 1.01 | 0.99 | 0.96 | 1.02 |
| Gender (F) | 1.42 | 0.76 | 2.64 | 1.15 | 0.58 | 2.29 |
| IT | 1.01 | 0.94 | 1.09 | 0.91 | 0.83 | 1.01 |
| CT | 1.16* | 1.07 | 1.25 | 1.21* | 1.08 | 1.35 |
| HT | 1.00 | 0.93 | 1.07 | 1.02 | 0.94 | 1.11 |
| AT | 1.04 | 0.99 | 1.10 | 1.00 | 0.92 | 1.07 |
| DT | 1.06 | 0.97 | 1.17 | 1.02 | 0.91 | 1.15 |

*: significant at 0.05 level
